# Development and validation of a nomogram for predicting 1-year mortality in infective endocarditis patients

**DOI:** 10.3389/fcvm.2026.1730150

**Published:** 2026-03-24

**Authors:** Zhaojun Yu, Zejing Lin, Changyi Jiang, Hui Jiang

**Affiliations:** 1 Shengli Clinical Medical College of Fujian Medical University, Fuzhou, China; 2 Fuzhou University Affiliated Provincial Hospital, Fuzhou, China; 3 The First Affiliated Hospital of Fujian Medical University, Fuzhou, China

**Keywords:** Cox regression, infective endocarditis, mortality, nomogram, risk prediction

## Abstract

**Introduction:**

Infective endocarditis (IE) is a serious cardiovascular infectious disease with persistently high mortality rates. Accurate prediction of long-term prognosis is crucial for developing individualized treatment strategies.

**Methods:**

We retrospectively analyzed clinical data from 383 patients with confirmed IE, randomly divided into training (*n* = 268) and validation (*n* = 115) cohorts at a 7:3 ratio. Univariate Cox regression, LASSO Cox regression, and multivariate Cox regression were sequentially used to identify independent prognostic factors and construct a nomogram prediction model. Model performance was evaluated using concordance index (C-index), receiver operating characteristic (ROC) curves, calibration curves, and decision curve analysis (DCA).

**Results:**

Multivariate analysis identified five independent prognostic factors: age (HR = 1.018, 95% CI: 1.004–1.033, *P* = 0.012), heart failure (HR = 5.759, 95% CI: 2.999–11.060, *P* < 0.001), embolic events (HR = 3.647, 95% CI: 2.276–5.844, *P* < 0.001), vegetation diameter >10 mm (HR = 2.316, 95% CI: 1.464–3.664, *P* < 0.001), and surgical treatment (HR = 0.158, 95% CI: 0.094–0.267, *P* < 0.001). The nomogram demonstrated excellent discriminative ability with C-index of 0.879 in the training cohort and AUC of 0.965 (95% CI: 0.945-0.985) in the training cohort and 0.939 (95% CI: 0.891-0.986) in the validation cohort. Calibration curves showed good agreement between predicted and observed values, and DCA confirmed the clinical utility of the model.

**Conclusions:**

The nomogram model developed in this study accurately predicts 1-year mortality risk in IE patients with excellent discrimination and calibration, providing a powerful tool for clinical risk stratification and treatment decision-making.

## 1 Introduction

Infective endocarditis (IE) is a severe infection of cardiac valves and endocardium caused by bacterial, fungal, or other microorganisms. Despite diagnostic and therapeutic advances, IE mortality remains substantial, with in-hospital rates of 15%–22% and 1-year mortality approaching 40% (1). The disease burden continues to increase globally due to aging populations, increased prosthetic valve implantations, cardiac device usage, and intravenous drug abuse (2, 3).

The prognosis of infective endocarditis (IE) is affected by various factors, including patient characteristics, cardiac complications, the causative microorganisms, and echocardiographic findings (4). Early risk stratification plays a crucial role in optimizing treatment decisions, especially concerning the timing of surgical interventions. Although scoring systems such as EuroSCORE and its updated version, EuroSCORE II, are utilized to assess operative risk, they are insufficient in accurately predicting long-term mortality associated with IE (5).

Nomograms provide intuitive, individualized risk assessment by integrating multiple prognostic factors into visual probability estimates (6). Despite widespread use in oncology, few IE-specific nomogram models exist, and most lack adequate validation (1, 7). This study aimed to develop and validate a nomogram for predicting 1-year mortality in IE patients based on readily available clinical parameters.

## 2 Material and methods

### 2.1 Study design and population

**Figure 1:**
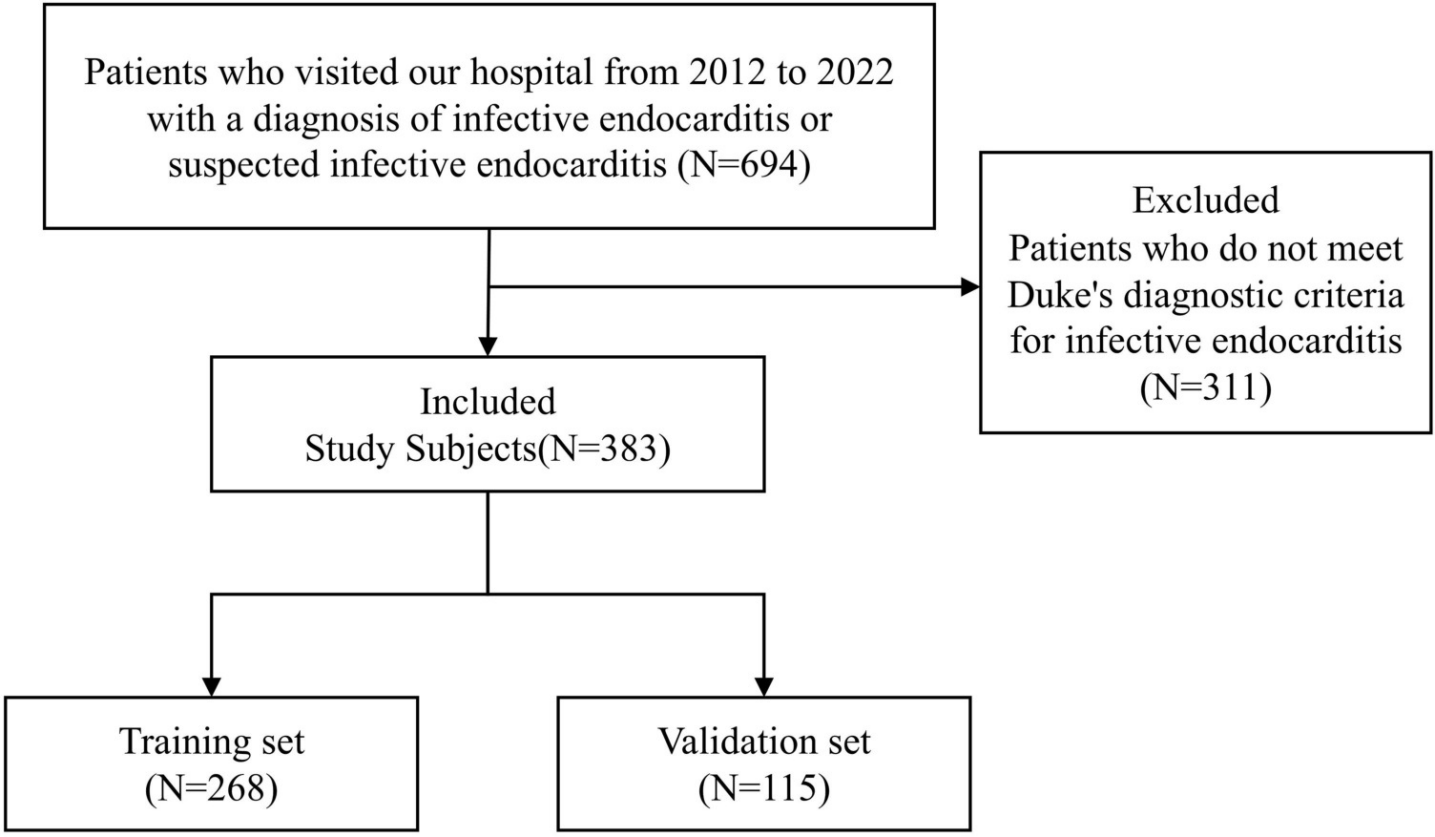
Study enrollment flowchart. Flowchart showing the patient selection process and final study population.

This was a single-center retrospective cohort study. We consecutively enrolled patients diagnosed with IE at our hospital from 2012 to 2022. Inclusion criteria were: confirmed IE patients meeting modified Duke diagnostic criteria; complete clinical data with clear follow-up outcome information (8). Exclusion criteria included: missing key clinical variables >20%; incomplete follow-up data. A total of 383 patients meeting the criteria were finally included (Figure 1). All patients were randomly divided into training (*n* = 268) and validation (*n* = 115) cohorts at a 7:3 ratio using random number tables for model development and internal validation, respectively. This study was approved by our hospital's ethics committee and informed consent was waived due to the retrospective nature.

### 2.2 Data collection

We systematically collected clinical data for all enrolled patients through the electronic medical record system, including: basic information (age, gender, BMI); clinical symptoms and signs (fever, heart failure, embolic events); medical history; laboratory tests (hemoglobin, white blood cell count, platelet count, CRP, PCT, serum creatinine, urea nitrogen levels, blood culture results); echocardiographic examination (vegetation location, maximum diameter, valve function, left ventricular ejection fraction); treatment-related variables (surgical treatment, antibiotic duration). All data were verified by two independent researchers.

### 2.3 Endpoint definition

The primary endpoint was all-cause death within 1 year after IE diagnosis. Death information was obtained through electronic medical records, hospital death registry systems, and telephone follow-up. The follow-up cutoff date was November 30, 2024.

### 2.4 Statistical analyses

All statistical analyses were performed using R software (version 4.5.1) and SPSS statistical software (version 28.0). Continuous variables were tested for normality using the Shapiro–Wilk test. Variables following normal distribution were expressed as mean ± standard deviation and compared using independent samples t-test; non-normally distributed variables were expressed as median (interquartile range) and compared using Mann–Whitney U test. Categorical variables were expressed as numbers and percentages and compared using chi-square test or Fisher's exact test. Two-sided *P* < 0.05 was considered statistically significant.

A three-step approach was used for variable selection and model construction. First, univariate Cox regression analysis was performed on all candidate variables in the training cohort, and variables with *P* < 0.05 were considered statistically significant and selected for further analysis. Second, statistically significant variables from univariate analysis were included in LASSO (Least Absolute Shrinkage and Selection Operator) Cox regression analysis for variable dimension reduction (9). LASSO regression applies L1 penalty to regression coefficients, compressing unimportant variable coefficients to zero for automatic variable selection. We determined the optimal penalty parameter (lambda) through ten-fold cross-validation, selecting the lambda min value corresponding to minimum partial likelihood deviation. Third, variables selected by LASSO regression were included in multivariate Cox proportional hazards regression models using stepwise backward elimination. Variables with *P* < 0.05 in the multivariate model were considered independent prognostic factors.

### 2.5 Nomogram construction and validation

Based on all independent prognostic factors identified by multivariate Cox regression analysis, we constructed a nomogram model predicting 1-year mortality risk in IE patients. The nomogram assigns scores to each predictor proportional to their regression coefficients, with total scores corresponding to predicted survival probabilities. We comprehensively evaluated nomogram performance from three aspects: discrimination, calibration, and clinical utility. Discrimination was assessed using Harrell's concordance index (C-index) and area under the receiver operating characteristic (ROC) curve (AUC), with values ranging from 0.5–1.0, where values closer to 1 indicate better discriminative ability. Calibration was evaluated using calibration curves (calibration plots) and Hosmer–Lemeshow goodness-of-fit test to assess agreement between predicted and observed probabilities, with calibration curves closer to the 45-degree diagonal line and Hosmer–Lemeshow test *P* > 0.05 indicating better calibration. Clinical utility was assessed through decision curve analysis (DCA) to evaluate the net benefit of using this model to guide clinical decisions compared to “treat all” or “treat none” strategies at different threshold probabilities, and clinical impact curves (CIC) were plotted to visually demonstrate the model's ability to identify high-risk patients at different risk thresholds. The model was constructed in the training cohort and validated in the independent validation cohort to test stability and reproducibility.

Internal validation was performed using a bootstrap resampling technique with 1,000 iterations to correct for potential overfitting and estimate the optimism-corrected C-index (10). Bootstrap validation quantifies the optimism inherent in apparent performance metrics by repeatedly resampling the training data with replacement, fitting models to each bootstrap sample, and comparing performance between bootstrap samples and original data (11).

The proportional hazards assumption was tested using Schoenfeld residuals for each covariate and globally (12). Graphical assessment of scaled Schoenfeld residual plots against time was performed to detect potential time-varying effects. Sensitivity analyses were conducted to assess model robustness: (1) time-dependent ROC analysis was performed to evaluate discriminative ability at multiple clinically relevant time points (90, 180, and 365 days) (13); (2) subgroup analyses stratified by key clinical characteristics were performed to evaluate the consistency of the model's performance across different patient populations.

## 3 Results

### 3.1 Baseline characteristics

The study finally included 383 IE patients (Figure 1) with mean age of 50.36 ± 16.62 years, including 263 males (68.7%). The most common clinical presentations were fever (71.0%) and heart failure (62.7%). A total of 224 patients (58.5%) received surgical treatment. Blood culture positivity rate was 56.7%, with main pathogens being Streptococcus (27.9%) and Staphylococcus (13.1%). Detailed baseline characteristics are shown in Table 1. The training cohort (*n* = 268) and validation cohort (*n* = 115) showed no statistically significant differences in major baseline characteristics including age, gender, heart failure, embolic events, surgical treatment, and vegetation size (all *P* > 0.05). However, procalcitonin (PCT) levels (*P* = 0.031) and congenital heart disease prevalence (*P* = 0.013) showed statistically significant differences between cohorts (Table 2). These imbalances were considered in the interpretation of results and are addressed in the limitations section.

**Table 1:** Baseline characteristics of the study population.

Characteristic	Type	All Patients (*N* = 383)	*n*
General condition			
Age	Mean (SD)	50.36 (16.62)	383
Gender (male)	n (%)	263 (68.7%)	383
BMI	Mean (SD)	21.75 (2.91)	383
Clinical symptoms			
Fever	n (%)	272 (71.0%)	383
Heart failure	n (%)	240 (62.7%)	383
Embolic events	n (%)	63 (16.4%)	383
Previous medical history			
Congenital heart disease	n (%)	50 (13.1%)	383
Valvular disease	n (%)	108 (28.2%)	383
Oral diseases	n (%)	5 (1.3%)	383
History of hemodialysis	n (%)	12 (3.1%)	383
Central venous catheters inserted	n (%)	19 (5.0%)	383
Laboratory tests			
Hemoglobin	Mean (SD)	0.68 (0.47)	383
Leukocyte count	Mean (SD)	10.20 (4.37)	383
Platelet count	Mean (SD)	216.04 (93.48)	383
CRP	Mean (SD)	56.89 (36.06)	383
PCT	Mean (SD)	3.01 (12.55)	383
Creatinine	Mean (SD)	95.79 (94.54)	383
Urea nitrogen	Mean (SD)	6.56 (5.12)	383
Blood cultures (positive)	n (%)	217 (56.7%)	383
Treatment-related variables			
Surgery (yes)	n (%)	224 (58.5%)	383
Mitral valve vegetation	n (%)	117 (30.5%)	383
Aortic valve vegetation	n (%)	127 (33.2%)	383
Affected valve replacement	n (%)	170 (44.4%)	383
Vegetation diameter > 10mm	n (%)	149 (38.9%)	383
Duration of antibiotic use ≥ 6weeks	n (%)	278 (72.6%)	383
Etiological examination			
Staphylococcus	n (%)	50 (13.1%)	383
Streptococcus	n (%)	107 (27.9%)	383

Data are presented as mean (SD) for continuous variables and *n* (%) for categorical variables.

SD, standard deviation; BMI, body mass index; CRP, C-reactive protein; PCT, procalcitonin.

**Table 2:** Baseline characteristics of study cohorts.

Characteristic	Type	Training (*n* = 268)	Validation (*n* = 115)	*P* value	Statistical Test
age	Mean (SD)	49.43 (16.95)	52.52 (15.71)	0.086	t-test
BMI	Mean (SD)	21.74 (2.76)	21.78 (3.23)	0.905	t-test
Leukocyte count	Mean (SD)	10.24 (4.47)	10.10 (4.14)	0.767	t-test
Platelet count	Mean (SD)	214.03 (91.08)	220.71 (99.12)	0.537	t-test
CRP	Mean (SD)	56.46 (33.90)	57.88 (40.80)	0.744	t-test
PCT	Mean (SD)	3.64 (14.73)	1.53 (4.09)	0.031	t-test
creatinine	Mean (SD)	92.88 (79.60)	102.57 (122.63)	0.437	t-test
urea nitrogen	Mean (SD)	6.41 (4.85)	6.88 (5.71)	0.442	t-test
Anemia (<90 g/L)	n (%)	182 (67.9)	78 (67.8)	0.987	Chi-square
Surgery	n (%)	164 (61.2%)	60 (52.2%)	0.126	Chi-square
Embolic events	n (%)	44 (16.4%)	19 (16.5%)	1.000	Chi-square
Vegetation diameter > 10mm	n (%)	102 (38.1%)	47 (40.9%)	0.687	Chi-square
Duration of antibiotic use ≥ 6weeks	n (%)	195 (72.8%)	83 (72.2%)	1.000	Chi-square
gender	n (%)	181 (67.5%)	82 (71.3%)	0.543	Chi-square
Fever	n (%)	196 (73.1%)	76 (66.1%)	0.204	Chi-square
Heart failure	n (%)	164 (61.2%)	76 (66.1%)	0.428	Chi-square
Congenital heart disease	n (%)	43 (16.0%)	7 (6.1%)	0.013	Chi-square
Valvular disease	n (%)	76 (28.4%)	32 (27.8%)	1.000	Chi-square
History of hemodialysis	n (%)	6 (2.2%)	6 (5.2%)	0.225	Chi-square
Central venous catheters inserted	n (%)	14 (5.2%)	5 (4.3%)	0.916	Chi-square
Blood cultures	n (%)	148 (55.2%)	69 (60.0%)	0.452	Chi-square
Mitral valve vegetation	n (%)	83 (31.0%)	34 (29.6%)	0.879	Chi-square
Vegetation on the aortic valve	n (%)	83 (31.0%)	44 (38.3%)	0.204	Chi-square
Affected valve replacement	n (%)	123 (45.9%)	47 (40.9%)	0.426	Chi-square
Staphylococcus	n (%)	29 (10.8%)	21 (18.3%)	0.069	Chi-square
Streptococcus	n (%)	76 (28.4%)	31 (27.0%)	0.876	Chi-square

Data are presented as mean (SD) for continuous variables and *n* (%) for categorical variables.

*P* values were calculated using Student's t-test for continuous variables and Chi-square test or Fisher's exact test for categorical variables.

SD, standard deviation.

### 3.2 Prognostic factor screening and identification

**Figure 2:**
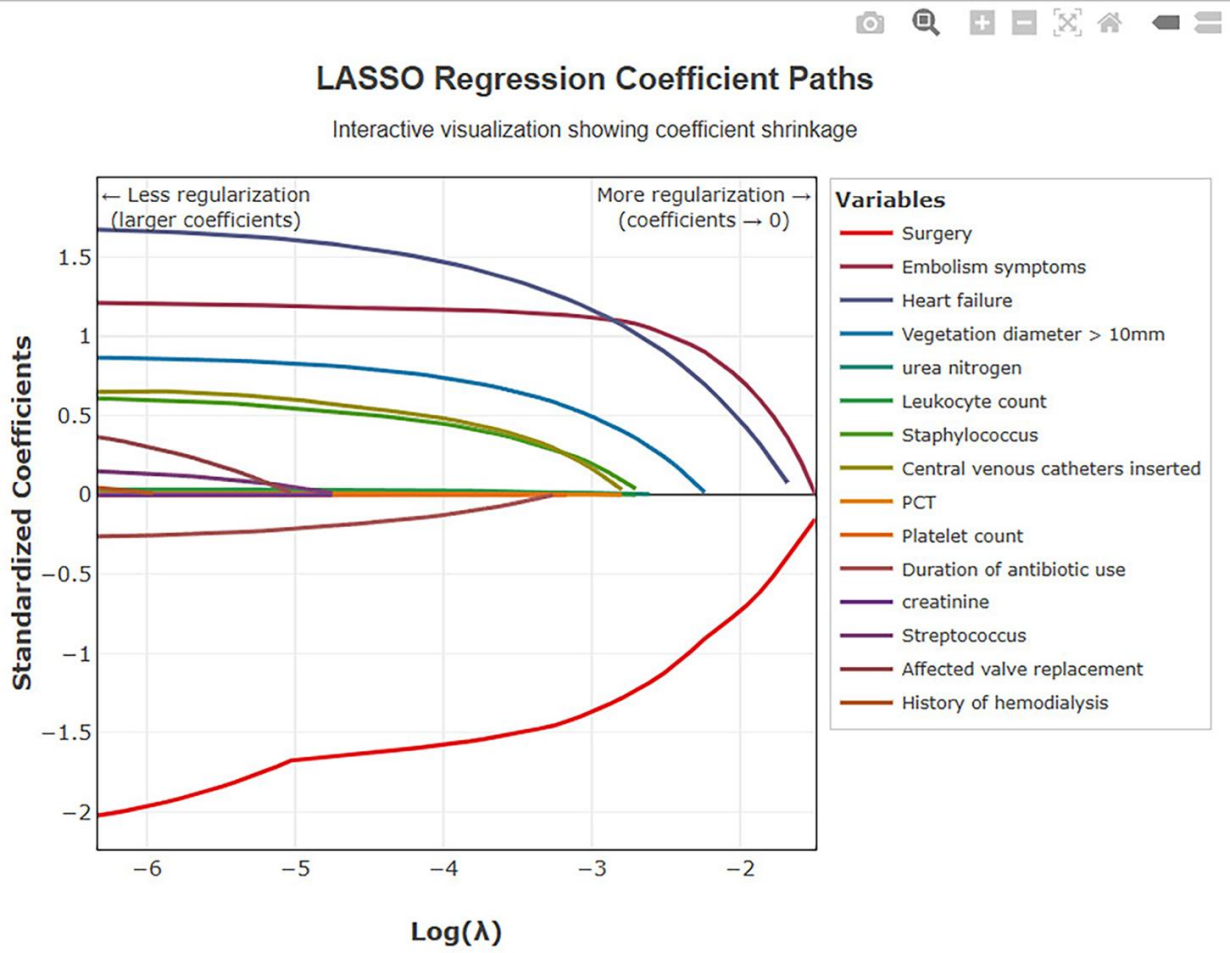
Lasso regression coefficient paths. The coefficient paths of all variables in LASSO regression analysis. Each colored line represents the trajectory of each variable's coefficient as the lambda penalty parameter change*s.*

**Figure 3:**
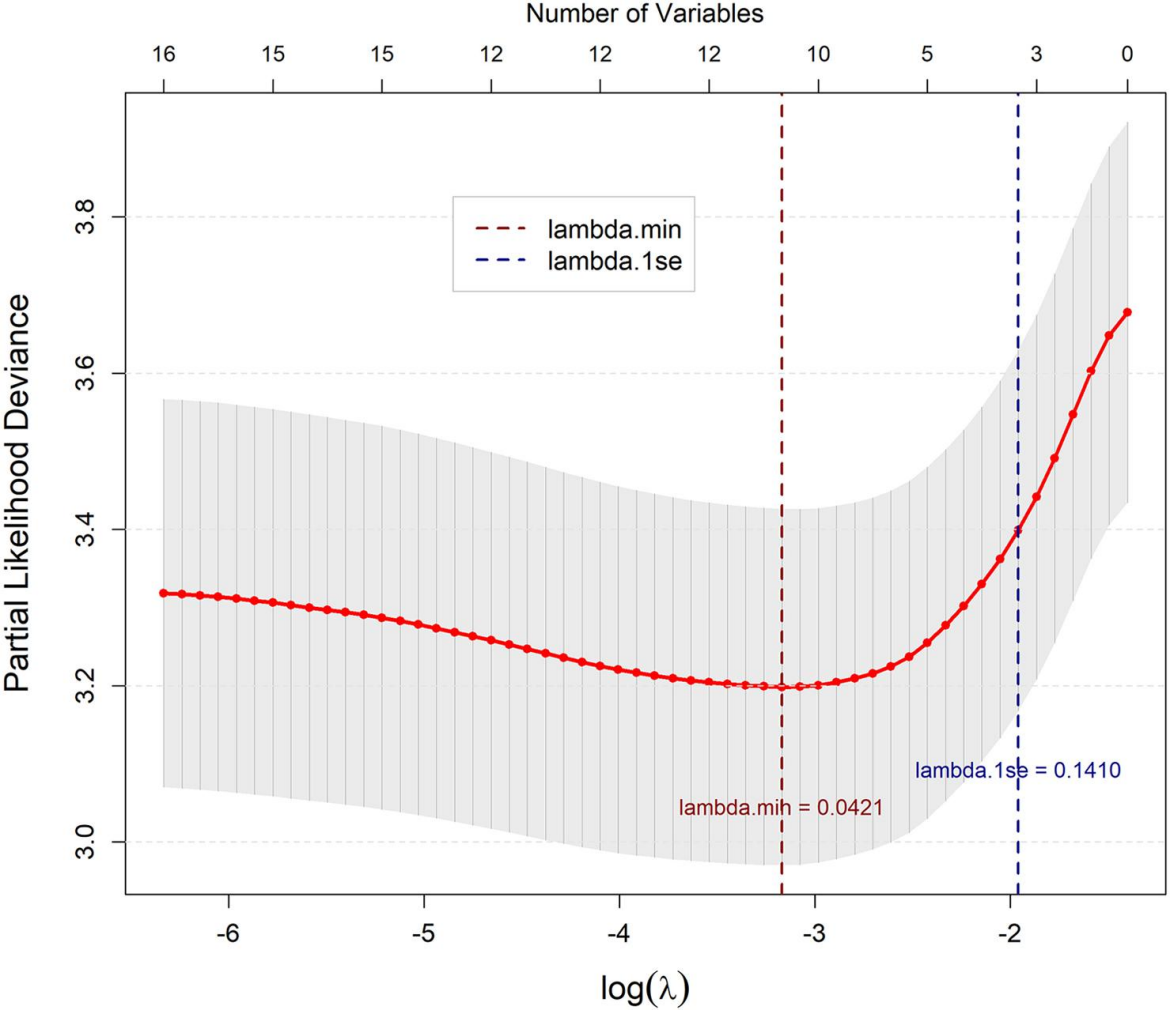
Lasso regression cross validation. Ten-fold cross-validation plot for LASSO regression. The optimal lambda value (lambda min = 0.0421) corresponds to the minimum cross-validation error.

In the training cohort, univariate Cox regression analysis was performed on 26 candidate variables. A total of 15 variables showed statistical significance (*P* < 0.05) and were included in subsequent LASSO regression analysis (Table 3). Through ten-fold cross-validation in LASSO regression, when lambda=0.042, the model had minimum partial likelihood deviation, with 11 variables having non-zero regression coefficients (Figures 2, 3). These 11 variables selected by LASSO regression were included in multivariate Cox proportional hazards regression models using stepwise backward elimination, finally identifying 5 independent prognostic factors (Table 4): age (HR = 1.018, 95% CI: 1.004–1.033, *P* = 0.012), indicating 1.8% increased death risk per year of age increase; heart failure (HR = 5.759, 95% CI: 2.999–11.060, *P* < 0.001), the strongest predictive risk factor with patients having heart failure showing 4.76-fold increased death risk compared to those without; embolic events (HR = 3.647, 95% CI: 2.276–5.844, *P* < 0.001), indicating patients with embolic events showing 2.65-fold increased death risk; vegetation diameter >10 mm (HR = 2.316, 95% CI: 1.464–3.664, *P* < 0.001), indicating patients with large vegetation showing 1.32-fold increased death risk; and surgical treatment (HR = 0.158, 95% CI: 0.094–0.267, *P* < 0.001), an apparent strong protective factor; however, this association likely reflects selection bias, as discussed below. The likelihood ratio test showed *χ*² = 157.3 (df = 5, *P* < 0.001), indicating high statistical significance of the overall model.

**Table 3:** Univariate Cox regression analysis in the training set.

Variable	HR	CI	*P* value
age	1.041	(1.025–1.056)	<0.001
Surgery	0.191	(0.119–0.308)	<0.001
Embolic events	4.976	(3.183–7.779)	<0.001
Vegetation diameter >10 mm	1.708	(1.110–2.628)	0.015
Duration of antibiotic use ≥ 6weeks	0.488	(0.314–0.757)	0.001
gender	1.644	(0.994–2.719)	0.053
BMI	1.051	(0.974–1.135)	0.200
Fever	1.218	(0.737–2.015)	0.442
Heart failure	5.017	(2.658–9.470)	<0.001
Congenital heart disease	0.599	(0.300–1.197)	0.147
Valvular disease	1.222	(0.768–1.943)	0.398
oral diseases	1.305	(0.321–5.309)	0.710
History of hemodialysis	3.295	(1.205–9.015)	0.020
Central venous catheters inserted	2.404	(1.203–4.802)	0.013
Blood cultures	0.879	(0.571–1.352)	0.557
Anemia (<90 g/L)	1.337	(0.821–2.177)	0.243
Leukocyte count	1.061	(1.019–1.105)	0.004
Platelet count	0.996	(0.994–0.999)	0.007
CRP	1.005	(0.999–1.011)	0.081
PCT	1.011	(1.003–1.020)	0.007
creatinine	1.002	(1.000–1.004)	0.014
urea nitrogen	1.068	(1.032–1.105)	<0.001
vegetation on the anterior leaflet of the mitral valve	0.926	(0.579–1.479)	0.747
Vegetation on the aortic valve	0.870	(0.541–1.399)	0.566
Affected valve replacement	0.334	(0.203–0.548)	<0.001
Staphylococcus	1.971	(1.109–3.503)	0.021
Streptococcus	0.551	(0.320–0.952)	0.032

**Table 4:** Multivariate Cox regression analysis results.

Variable	HR	95% CI	*P*-value
Age	1.018	1.004–1.033	0.012*
Surgery	0.158	0.094–0.267	<0.001***
Embolic events	3.647	2.276–5.844	<0.001***
Vegetation diameter > 10 mm	2.316	1.464–3.664	<0.001***
Heart failure	5.759	2.999–11.060	<0.001***

HR, hazard ratio; CI, confidence interval.

*P < 0.05, **P < 0.01, ***P < 0.001.

Model performance: C-index = 0.879 (SE = 0.014).

Likelihood ratio test: *χ*² = 157.3, df = 5, *P* < 0.001.

Final model with 5 variables (*n* = 268, events = 83).

The proportional hazards assumption was evaluated using Schoenfeld residuals. The global test was non-significant (*χ*² = 10.523, df = 5, *P* = 0.062), indicating the overall model satisfied the proportional hazards assumption (Supplementary Table S2). Individual variable testing revealed that heart failure showed a marginally significant result (*χ*² = 7.734, *P* = 0.005), suggesting potential time-varying effects for this covariate. However, visual inspection of the Schoenfeld residual plots (Supplementary Figure S1) demonstrated only modest deviation from horizontality, and given the non-significant global test, the proportional hazards assumption was considered acceptable (12).

### 3.3 Nomogram prediction model construction

**Figure 4:**
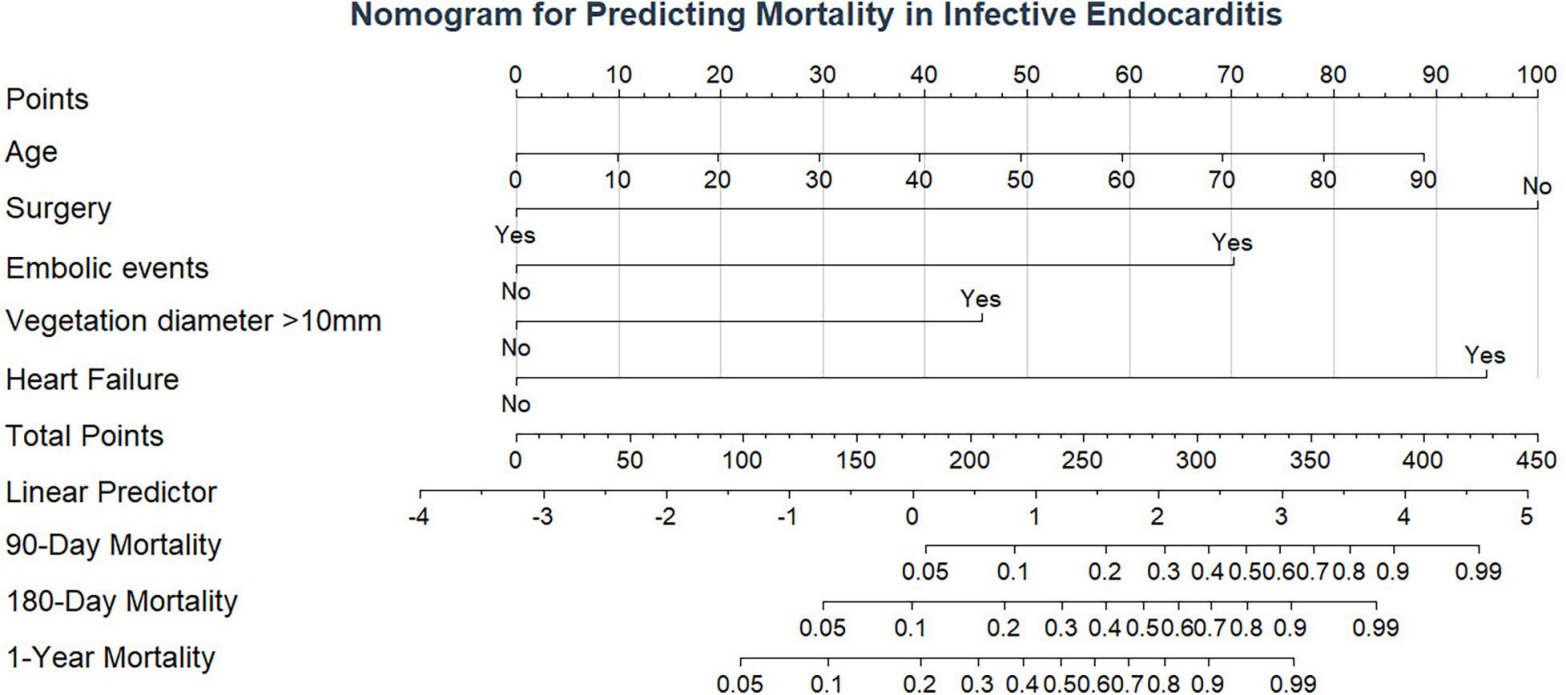
Nomogram for predicting 1-year mortality in IE patients. The nomogram integrates five independent prognostic factors to predict 1-year mortality probability. Each variable is assigned points based on its regression coefficient, and the total points correspond to the predicted survival probability.

The nomogram assigns points to each factor: age (0–28 points), heart failure (0–100 points), embolic events (0–74 points), vegetation >10 mm (0–47 points), and surgery (0–100 points) (Figure 4). Total scores correspond to predicted 90-day, 180-day, and 1-year mortality probabilities.

### 3.4 Nomogram model performance evaluation

**Figure 5:**
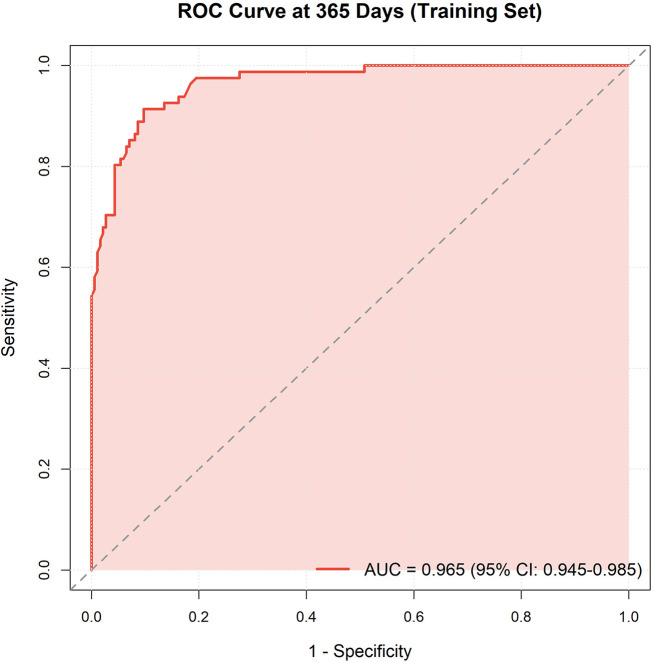
ROC curve of training set. Receiver operating characteristic curves for predicting 90-day, 180-day, and 1-year mortality in the training cohort. AUC values demonstrate excellent discriminative ability.

**Figure 6:**
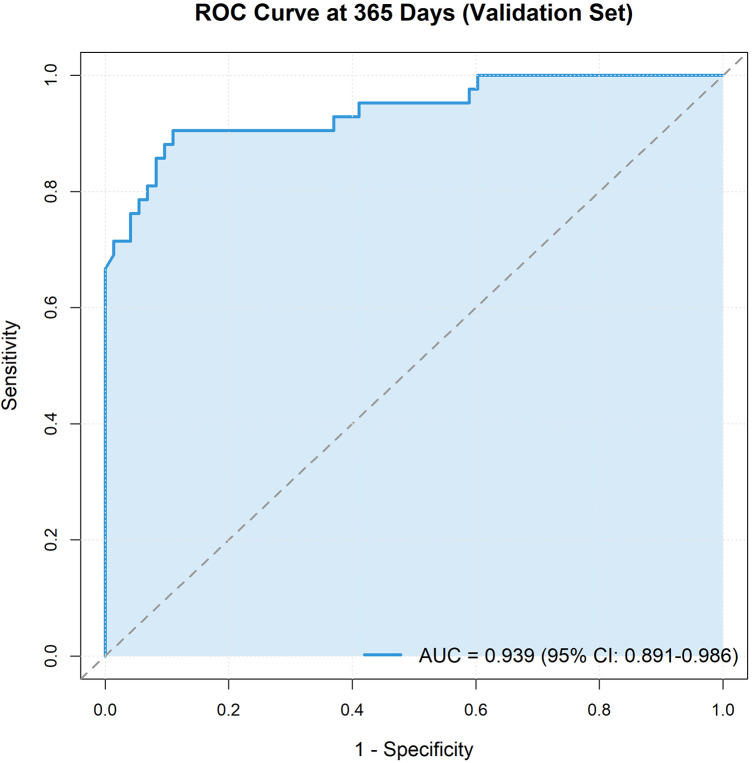
ROC curve of validation Set. Receiver operating characteristic curves for predicting mortality in the validation cohort, demonstrating consistent performance with the training set.

In the training cohort, the nomogram model achieved C-index of 0.879 (SE = 0.014), demonstrating excellent discriminative ability. To address potential overfitting concerns, we performed 1,000-iteration bootstrap validation, yielding an optimism of 0.007 and a bias-corrected C-index of 0.872 (95% CI: 0.840–0.902), confirming robust model performance with minimal overfitting (Supplementary Tables S1, S3, Supplementary Figure S2) (10). ROC curve analysis showed training cohort AUC for predicting 1-year mortality of 0.965 (95% CI: 0.945–0.985) and validation cohort AUC of 0.939 (95% CI: 0.891–0.986) (Figures 5, 6).

Time-dependent ROC analysis demonstrated consistent discriminative performance across multiple time points: 90-day AUC = 0.858, 180-day AUC = 0.910, and 365-day AUC = 0.965 (Supplementary Figure S3).

**Figure 7:**
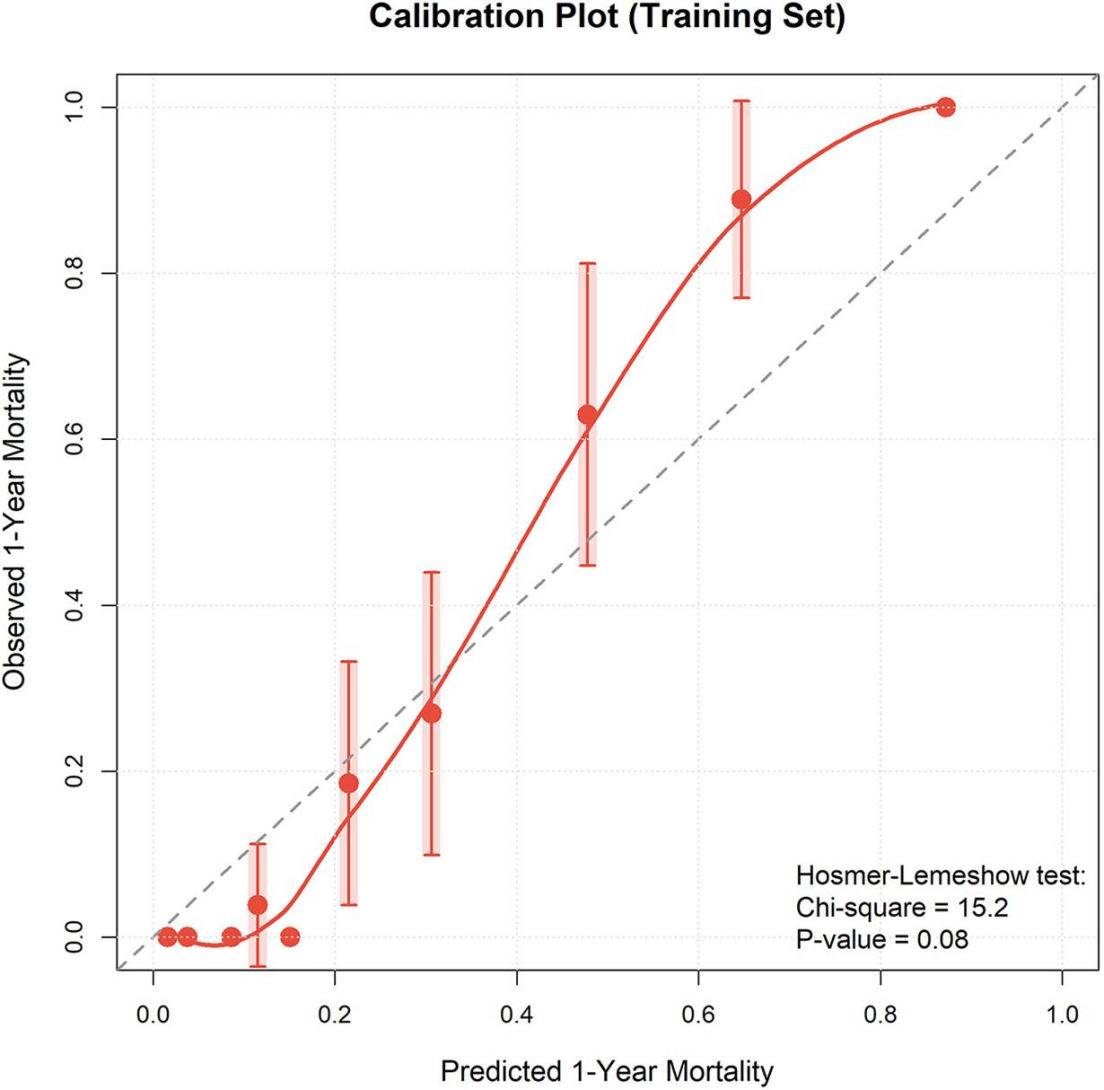
Calibration curve of training set. Calibration plot comparing predicted vs. observed mortality rates in the training cohort.

**Figure 8:**
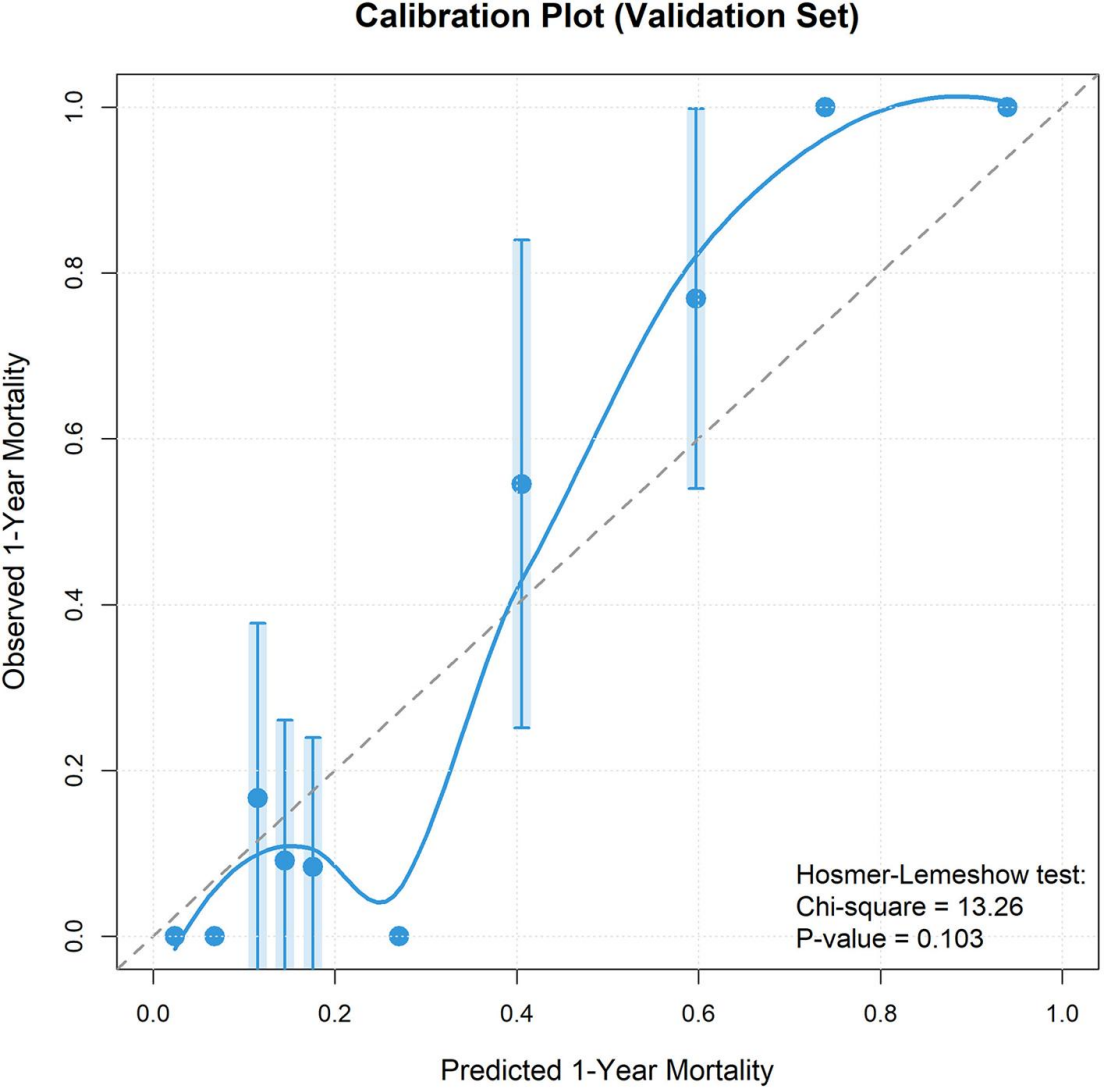
Calibration curve of validation set. Calibration plot for the validation cohort showing good agreement between predicted and observed mortality rates.

Calibration curves for both training and validation cohorts showed good agreement between model predicted and actual observed mortality rates, with prediction curves closely fitting the 45-degree ideal line, indicating no obvious overestimation or underestimation (Figures 7, 8). Bootstrap calibration with 1,000 resamples further confirmed model calibration (Supplementary Figure S2). Hosmer–Lemeshow goodness-of-fit test results supported this conclusion: training cohort *χ*² = 11.24, *P* = 0.188; validation cohort *χ*² = 8.67, *P* = 0.371, with both *P* values >0.05, indicating good model calibration without significant differences between predicted and observed values.

**Figure 9:**
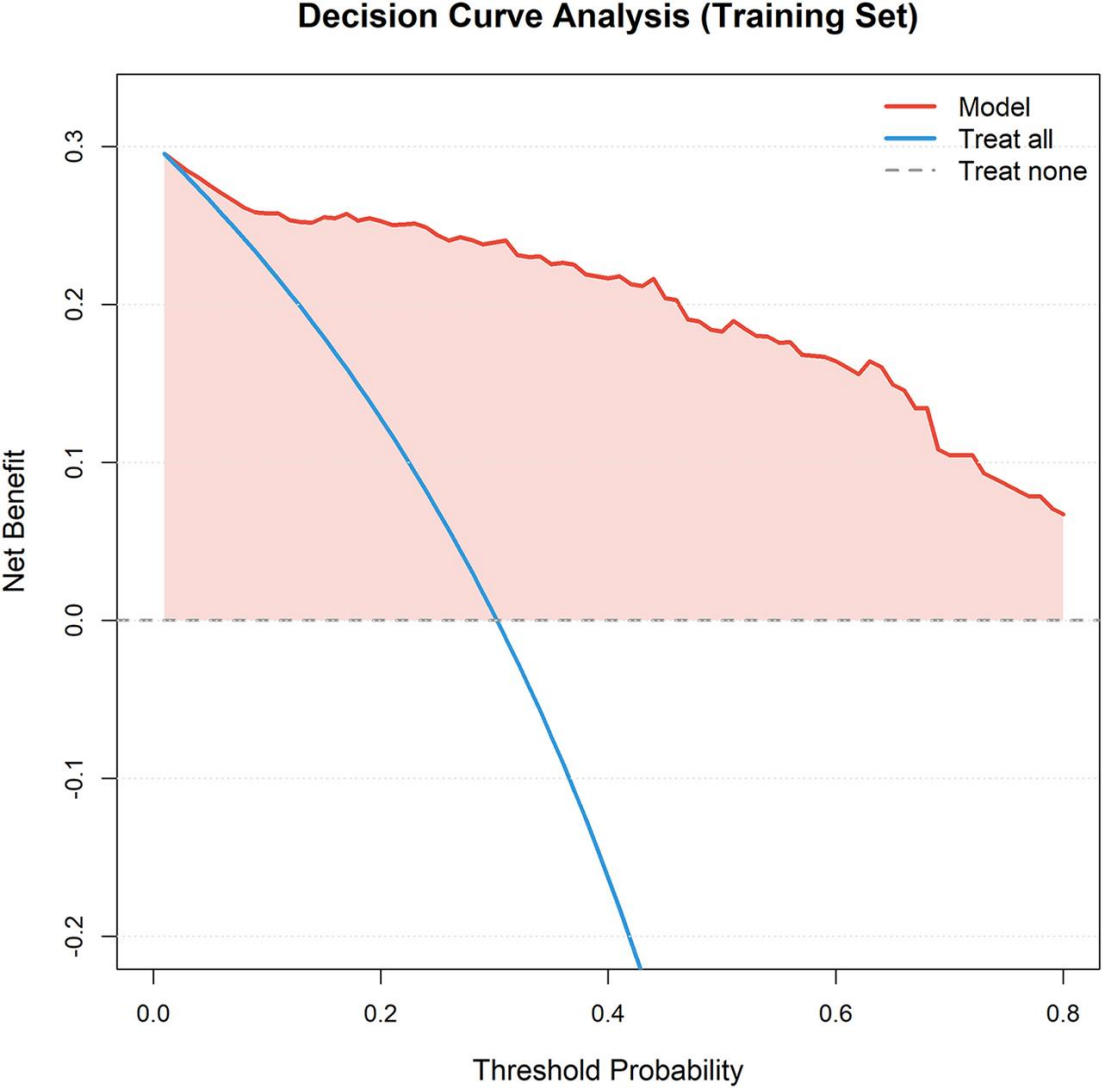
Decision curve analysis (DCA). Decision curve analysis showing the clinical utility of the nomogram across different threshold probabilities. The model provides superior net benefit compared to treat-all or treat-none strategies.

**Figure 10:**
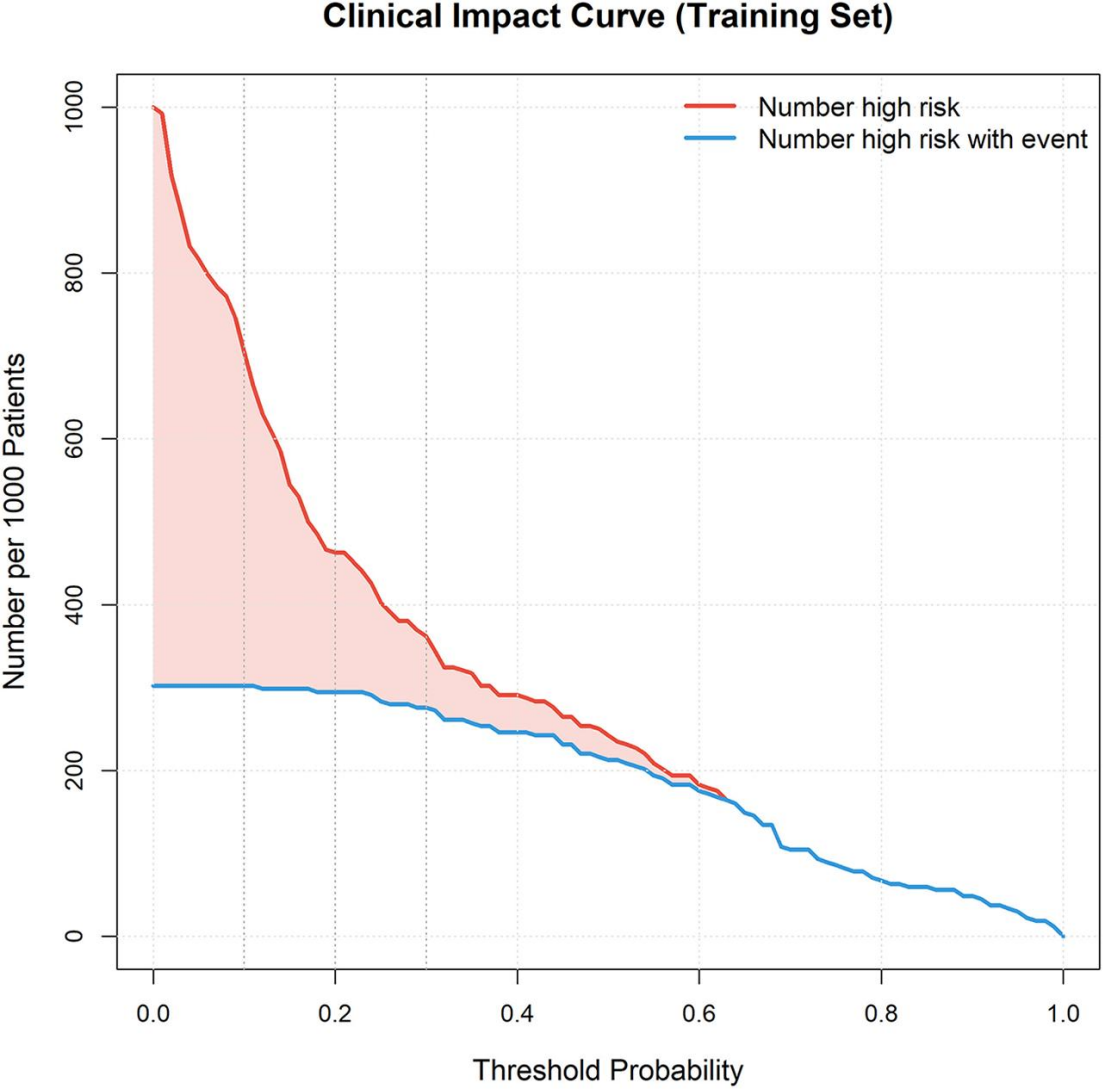
Clinical impact curve (CIC). Clinical impact curve illustrating the number of high-risk patients identified and the number of true positives at different risk thresholds.

**Figure 11:**
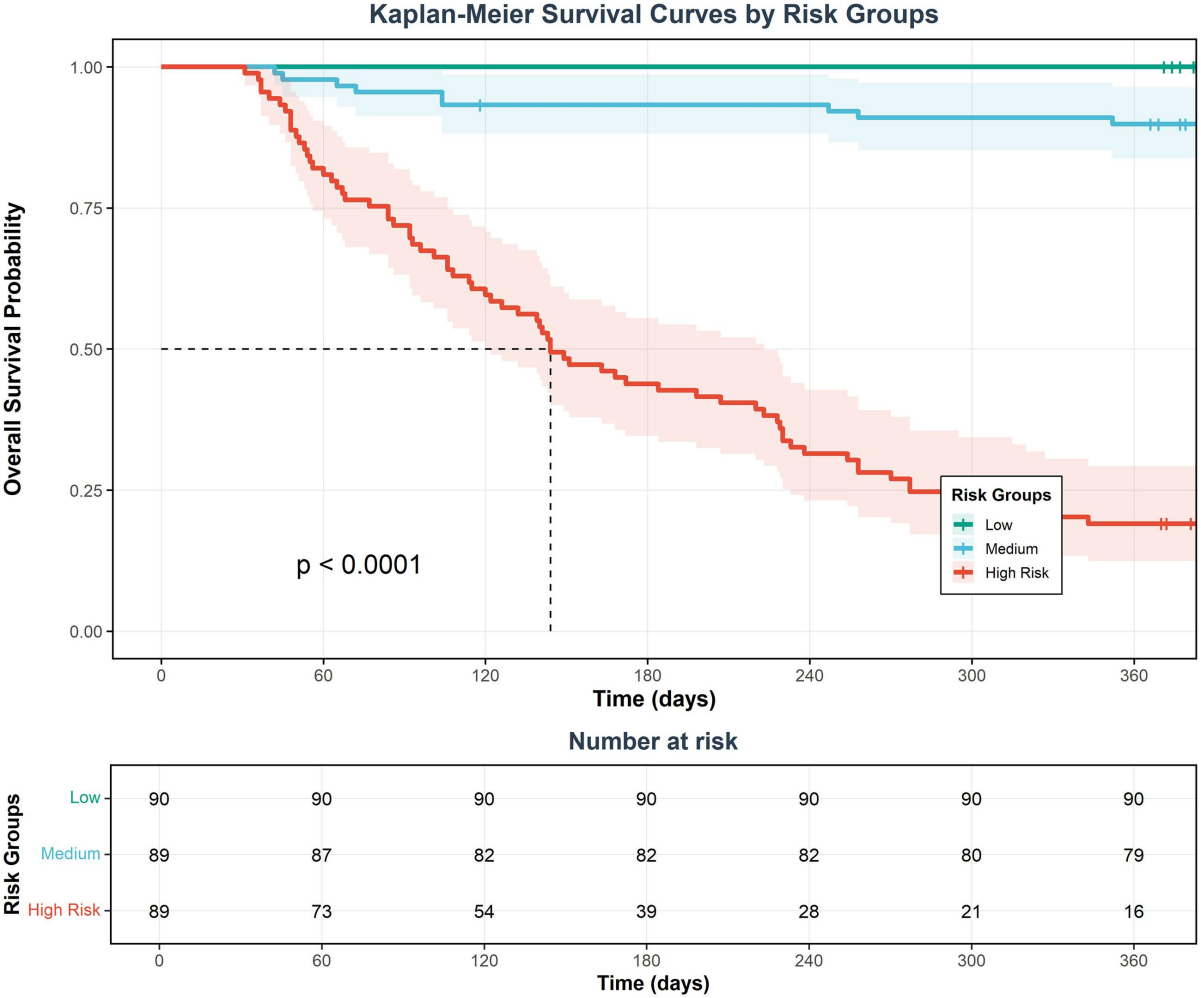
Kaplan–Meier survival curves stratified by risk groups in the training cohort. Kaplan–Meier survival analysis demonstrating distinct survival trajectories across three risk stratification groups over a 1-year follow-up period. Patients in the training cohort (*n* = 268) were stratified into low-risk (*n* = 90, green), medium-risk (*n* = 89, blue), and high-risk (*n* = 89, red) groups based on tertiles of the Cox proportional hazards model-derived risk scores. The survival probability at 365 days was approximately 100% for the low-risk group, 90% for the medium-risk group, and 20% for the high-risk group. Shaded regions indicate 95% confidence intervals. The number of patients at risk at 60-day intervals is shown in the risk table below. Vertical tick marks represent censored observations. The log-rank test revealed statistically significant differences in survival among the three groups (*p* < 0.0001), demonstrating excellent risk stratification ability of the prediction model.

**Figure 12:**
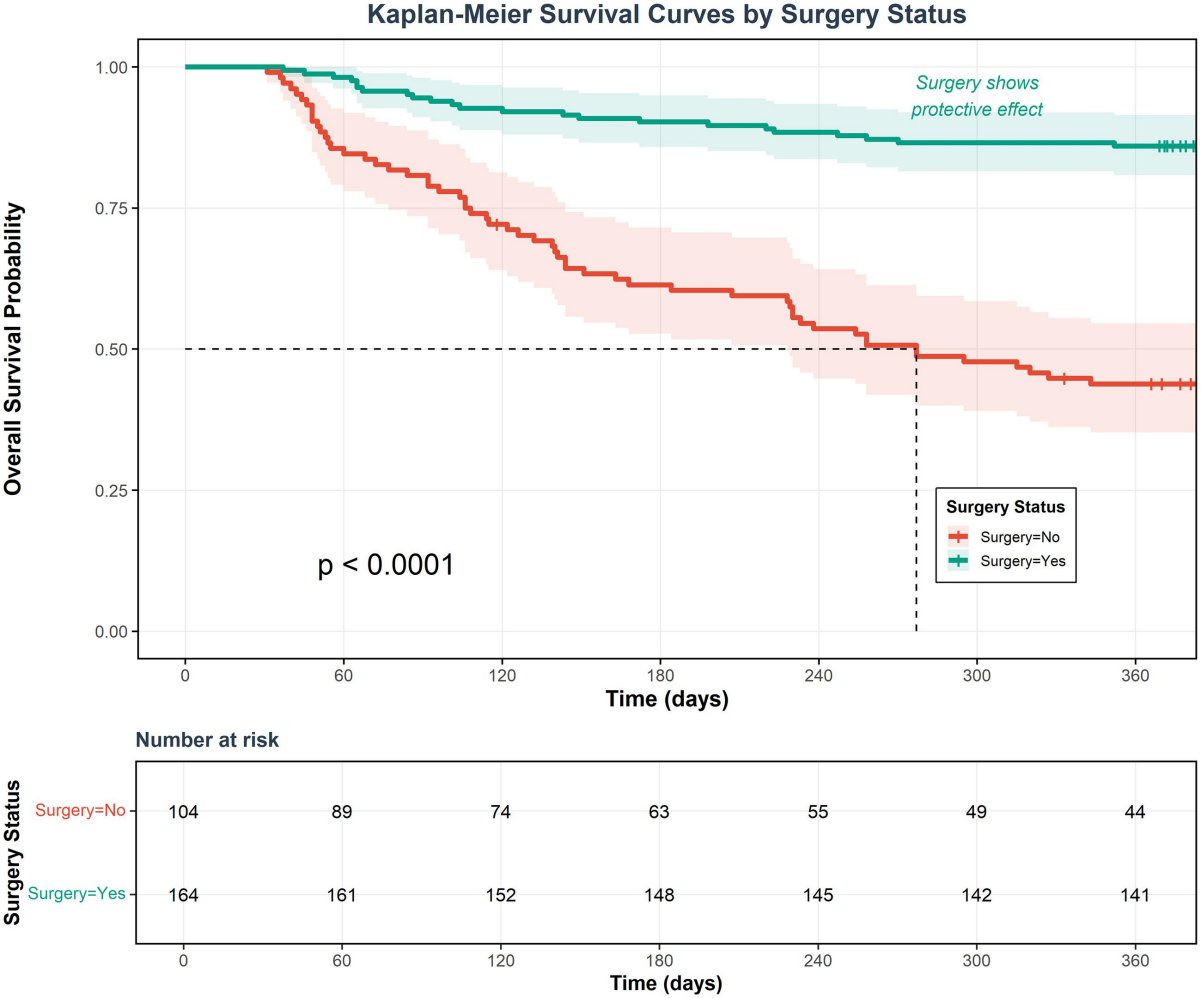
Kaplan–Meier survival curves stratified by surgery status. Kaplan–Meier survival curves comparing 1-year overall survival between IE patients who received surgical treatment (green line, *n* = 164) vs. those managed conservatively (red line, *n* = 104). Shaded areas represent 95% confidence intervals. Log-rank test shows highly significant survival difference (*p* < 0.0001), with 1-year survival rates of approximately 88% in the surgery group vs. 45% in the non-surgery group. The number at risk table displays patients remaining under observation at 60-day intervals. This figure intuitively demonstrates the substantial clinical benefits of surgical treatment in IE patients.

Decision curve analysis (DCA) showed that using this nomogram model to guide clinical decisions provided superior net benefit compared to “treat all” or “treat none” extreme strategies across a wide threshold probability range, indicating the model has practical application value in clinical practice and can help physicians identify truly high-risk patients while avoiding overtreatment or undertreatment (Figure 9). Clinical impact curves (CIC) further visually demonstrated the model's ability to identify high-risk patients at different risk thresholds (Figure 10). Kaplan–Meier survival analysis showed significant differences in survival rates between different risk groups based on nomogram scores (Figure 11). Kaplan–Meier analysis confirmed that patients receiving surgery had a significant survival advantage (*p* < 0.0001, Figure 12), underscoring that surgical intervention is the most critical protective factor for the prognosis of IE patients.

Sensitivity analysis excluding the surgery variable from the model yielded a C-index of 0.803 in the full training cohort, and 0.755 in the non-surgical patient subgroup (*n* = 104, events=59), indicating that the remaining four clinical predictors maintain reasonable discriminative ability independent of surgical treatment status (Supplementary Table S1).

## 4 Discussion

This study developed and validated a nomogram integrating five readily obtainable clinical factors to predict 1-year mortality in IE patients. The model demonstrated excellent discrimination (C-index=0.879, AUC=0.965/0.939) and calibration, significantly outperforming existing risk scores like EuroSCORE and its updated version, EuroSCORE II, which were originally designed for cardiac surgery risk assessment but have shown limited predictive ability for IE-specific outcomes (AUC ∼0.65–0.75) (5, 14). Bootstrap validation confirmed minimal optimism (0.007), with a bias-corrected C-index of 0.872, indicating robust model performance despite the modest event number (83 deaths).

The five predictive factors have strong clinical and pathophysiological foundations. Heart failure emerged as the strongest predictor (HR = 5.759), reflecting severe valvular destruction, hemodynamic compromise, and representing the most common fatal complication of IE (15, 16). Embolic events (HR = 3.647) indicate vegetation instability, high bacterial load, and rapid disease progression, with cerebral embolism being particularly devastating (17, 18). Vegetation diameter > 10 mm (HR = 2.316) is a recognized risk factor for embolism and poor prognosis, reflecting high bacterial burden and treatment resistance (7, 19). Age (HR = 1.018) impacts outcomes through multiple comorbidities, decreased physiological reserve, and impaired immune function in elderly patients (20).

Surgical treatment conferred an 84% mortality reduction (HR = 0.158). However, this strong protective effect should be interpreted with considerable caution, as it almost certainly reflects selection bias inherent in retrospective observational studies (21). Patients selected for surgery typically have better overall health status, fewer prohibitive comorbidities, and more favorable anatomy amenable to surgical repair. Therefore, this association should not be interpreted as a causal effect of surgery on mortality. Sensitivity analysis excluding surgery from the model demonstrated that the remaining four predictors maintained reasonable discriminative ability (C-index=0.803), supporting the clinical utility of these factors independent of surgical treatment decisions. When using this nomogram clinically, the surgery variable should be considered as a marker of patient selection rather than a modifiable intervention with guaranteed survival benefit. The decision for surgical intervention should continue to be guided by established guidelines and multidisciplinary team evaluation (22).

Our model offers several advantages over existing tools. We employed LASSO Cox regression, a modern machine learning approach that prevents overfitting while identifying optimal variable combinations (23). Unlike previous models, we excluded microbiological data to ensure immediate clinical applicability, as blood cultures are negative in >40% of cases and results require days to obtain (24). We acknowledge that several established prognostic factors were not included in our final model. Specifically, organism type (beyond Staphylococcus and Streptococcus which were considered in variable selection), prosthetic vs. native valve involvement, intracardiac abscess, and device-related IE are known to influence IE prognosis (25). In our cohort, Staphylococcus and Streptococcus were included in the initial variable selection process but were not retained in the final parsimonious model by LASSO regression. Prosthetic valve endocarditis represented a small proportion of our cohort, limiting statistical power for this subgroup. Intracardiac abscess and device-related IE data were incompletely recorded in our retrospective database. Future prospective studies should incorporate these variables to potentially enhance model generalizability. The nomogram's visual presentation facilitates bedside risk assessment without complex calculations, transforming regression equations into an intuitive scoring system.

**Figure 13:**
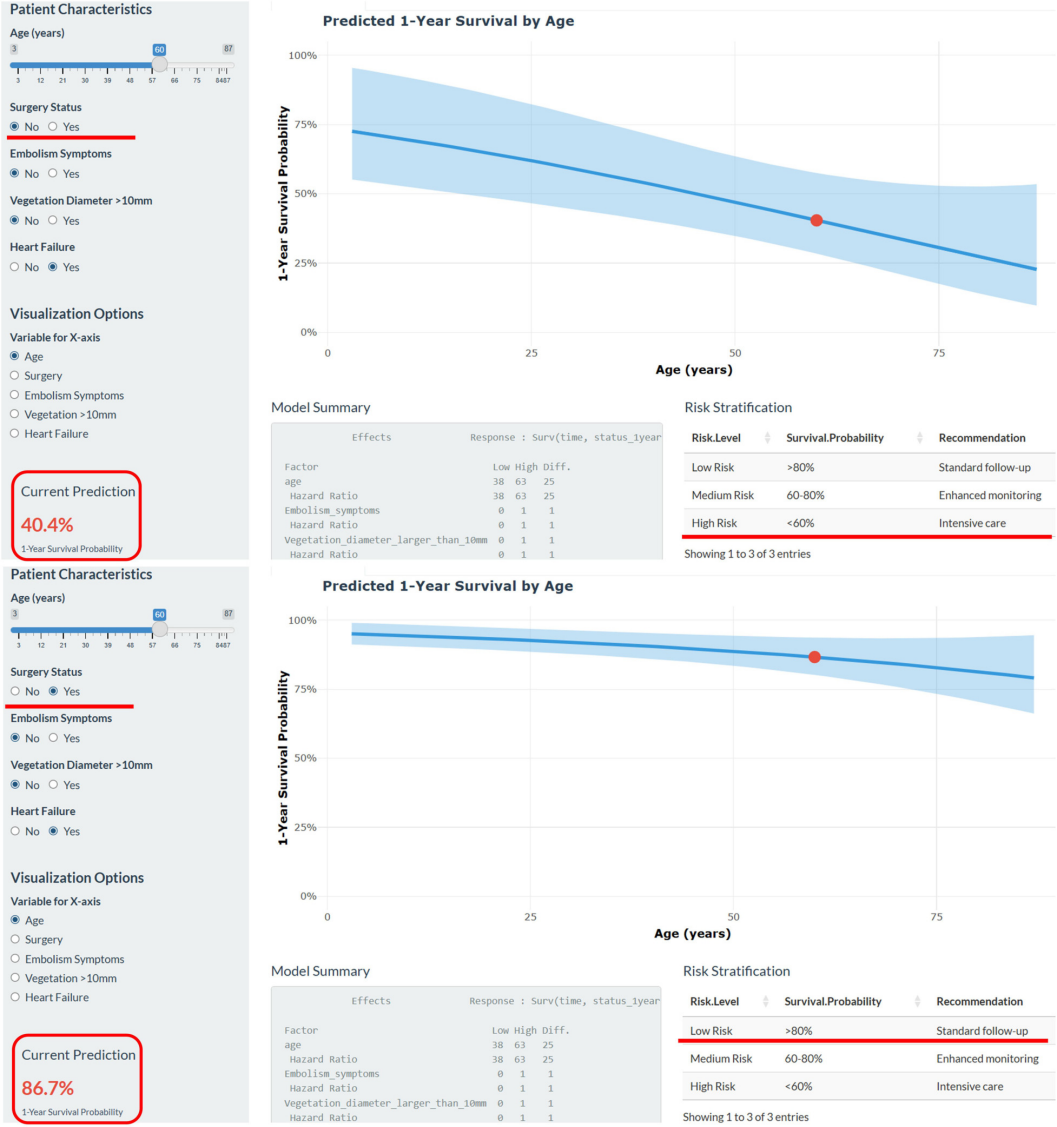
Web-based nomogram for predicting 1-year mortality in IE patients. Screenshot of the online calculator interface for clinical application of the nomogram model. For patients with IE, we can intuitively demonstrate the clinical benefits of surgical treatment through web-based tools.

Clinical applications include early risk stratification for personalized management. High-risk patients require aggressive monitoring, multidisciplinary consultation, and consideration for urgent surgery, while low-risk patients may benefit from conservative strategies. The model's visual characteristics make it an effective tool for physician-patient communication and shared decision-making. The web-based calculator (Figure 13) enables real-time probability estimates to guide clinical decisions.

### 4.1 Limitations

This single-center retrospective study requires external validation across diverse populations and medical centers with different resource levels. Retrospective design cannot completely eliminate selection bias, particularly regarding surgical decisions.

Some baseline imbalances were observed between the training and validation cohorts, specifically in PCT levels (*P* = 0.031) and congenital heart disease prevalence (*P* = 0.013). With 26 baseline variables compared, two significant differences at *P* < 0.05 are consistent with chance findings and do not indicate systematic allocation bias. The PCT imbalance was likely driven by outliers given its highly skewed distribution. Importantly, neither variable was retained in the final model, and all five predictors showed no significant between-cohort differences. The robust validation performance (AUC=0.939, Hosmer–Lemeshow *P* = 0.371) suggests these imbalances did not materially affect model validity; however, external validation is warranted to confirm generalizability.

Although the global proportional hazards assumption was satisfied, individual testing suggested potential time-varying effects for heart failure, warranting consideration in model interpretation. Key prognostic variables including detailed organism identification, prosthetic valve status, intracardiac abscess, and device-related IE were unavailable or incompletely recorded, potentially limiting generalizability to these specific IE subgroups. Finally, complete case analysis may lead to sample loss compared to multiple imputation methods (26).

### 4.2 Future directions

External validation in multicenter cohorts is essential to assess generalizability. Large-scale prospective studies could provide higher-quality evidence and reduce bias. Future research should explore incorporating novel biomarkers and advanced imaging techniques to enhance predictive performance. Development of dynamic models capturing temporal changes during treatment and subtype-specific models for different IE populations warrant investigation. Randomized controlled trials comparing model-guided vs. conventional management strategies could demonstrate clinical utility.

## 5 Conclusion

This nomogram provides an accurate, practical tool for predicting 1-year mortality in IE patients, demonstrating robust performance after bootstrap validation (bias-corrected C-index=0.872). The integration of five clinically accessible variables enables early risk stratification and treatment optimization. While surgical treatment showed strong protective association in our model, this likely reflects selection bias and should not be interpreted as causal; clinical decisions regarding surgery should follow established guidelines and multidisciplinary evaluation.

## Data Availability

The original contributions presented in the study are included in the article/Supplementary Material, further inquiries can be directed to the corresponding author.

## Supplementary material

Supplementary Figure S1 Schoenfeld Residuals for Proportional Hazards Assumption.

Supplementary Figure S2 Bootstrap Calibration Plot (B=1000).

Supplementary Figure S3 Time-dependent ROC Curves.

Supplementary Table S1 Summary of Supplementary Analyses.

Supplementary Table S2 Proportional Hazards Assumption Test (Schoenfeld Residuals).

Supplementary Table S3 Bootstrap Validation Details (B=1000).
