# Supplementary figures and images for: Development and validation of a nomogram for predicting 1-year mortality in infective endocarditis patients
Source: Front Cardiovasc Med. 2026 Mar 24;13:1730150. doi: 10.3389/fcvm.2026.1730150 (PMC13053318; doi:10.3389/fcvm.2026.1730150)

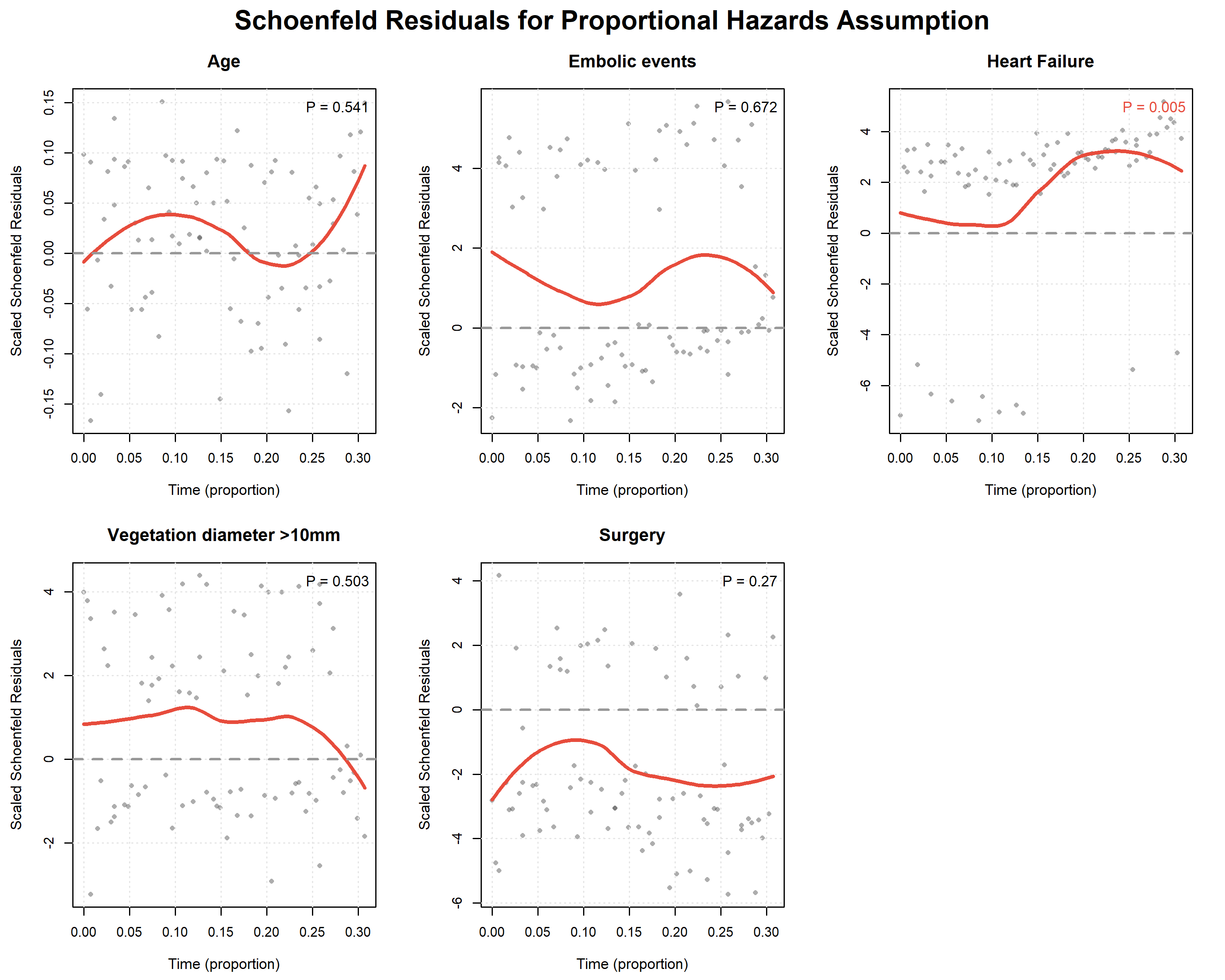

Supplement: Supplementary Figure S1 — Schoenfeld Residuals for Proportional Hazards Assumption. [file Image1.jpg]

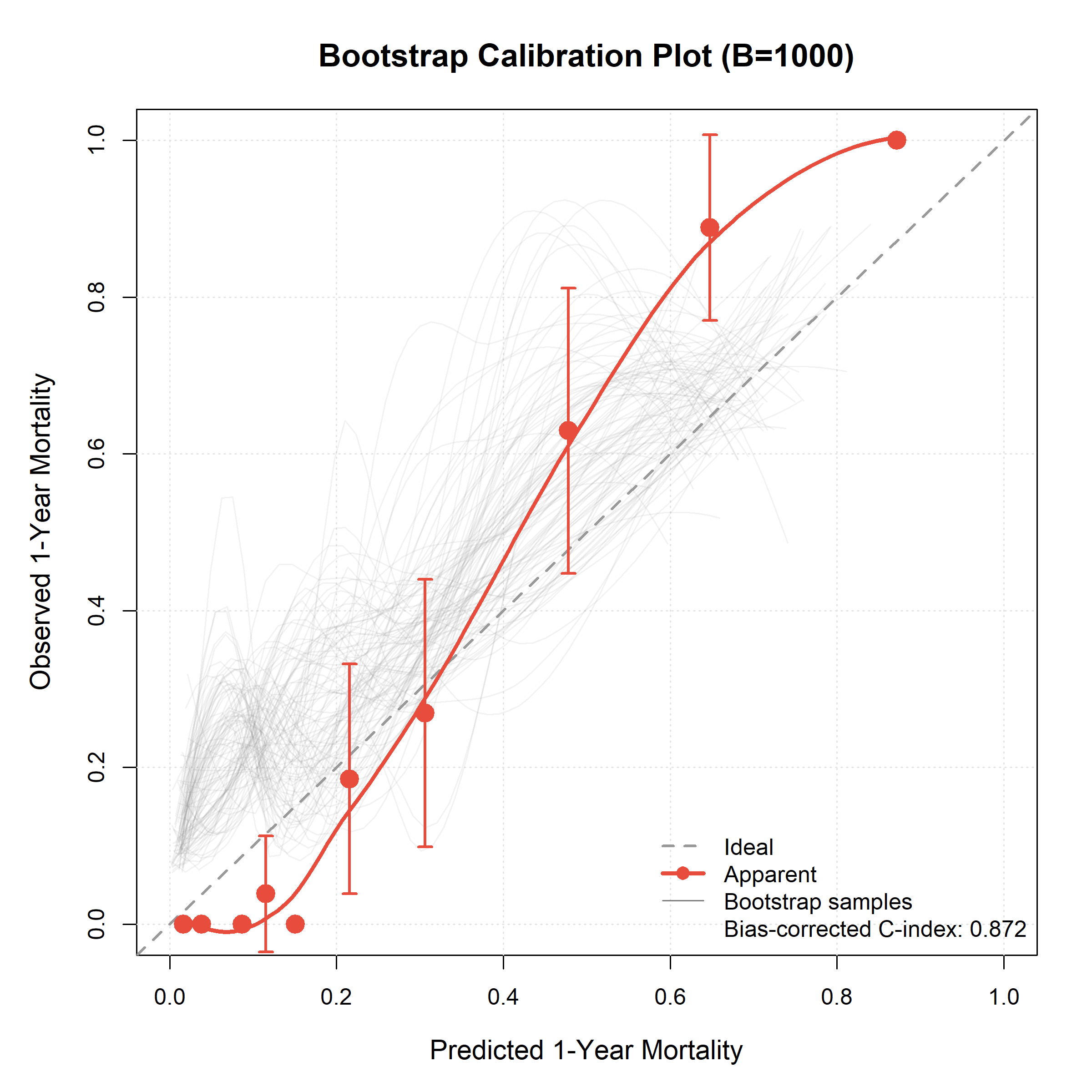

Supplement: Supplementary Figure S2 — Bootstrap Calibration Plot (B=1000). [file Image2.jpg]

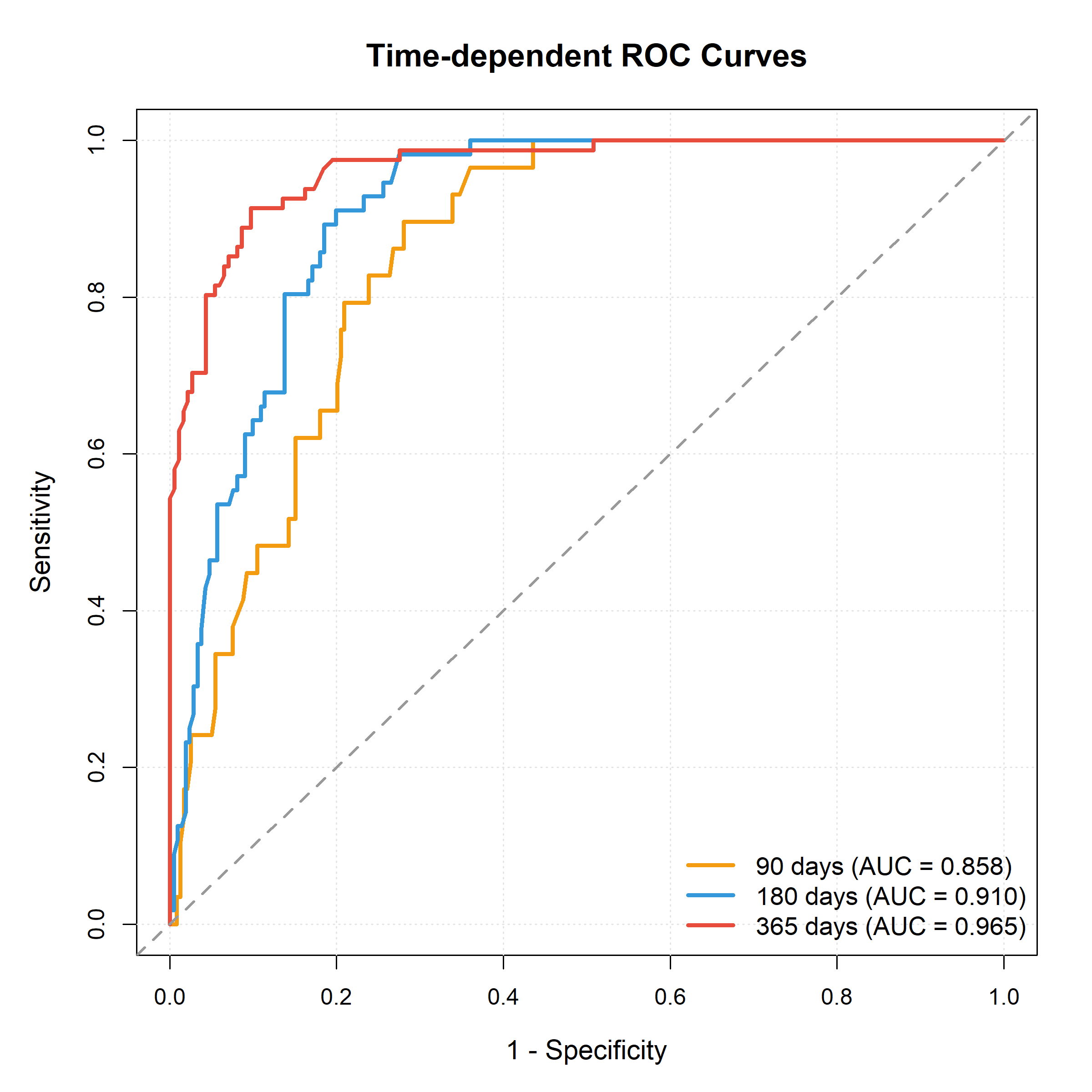

Supplement: Supplementary Figure S3 — Time-dependent ROC Curves. [file Image3.jpg]
