# Supplementary material for: Development and validation of a nomogram for predicting 1-year mortality in infective endocarditis patients
Source: Front Cardiovasc Med. 2026 Mar 24;13:1730150. doi: 10.3389/fcvm.2026.1730150 (PMC13053318; doi:10.3389/fcvm.2026.1730150)
Supplement: Supplementary Table S1 — Summary of Supplementary Analyses. [file Table1.docx]

**Supplementary Table S1. Summary of Supplementary Analyses**

Bootstrap validation, proportional hazards testing, and sensitivity analyses results.

| **Analysis Category** | **Metric** | **Value** |
| --- | --- | --- |
| **Primary Analysis** | **Original C-index** | **0.879** |
|  | Bootstrap corrected C-index (B=1000) | 0.872 |
|  | Optimism | 0.007 |
|  | 95% CI for corrected C-index | 0.840-0.902 |
|  | 365-day AUC | 0.965 |
| **Proportional Hazards Test** | **Global test χ²** | **10.523 (df=5)** |
|  | Global test P-value | 0.062 |
| **Sensitivity Analysis 1: Excluding early deaths** | **(<30 days)** |  |
|  | Sample size | 268 |
|  | Number of events | 83 |
|  | C-index | 0.879 |
| **Sensitivity Analysis 2: Different time points** |  |  |
|  | 90-day AUC | 0.858 |
|  | 180-day AUC | 0.910 |
|  | 365-day AUC | 0.965 |
| **Sensitivity Analysis 3: Excluding Surgery** | **variable** |  |
|  | C-index (all patients) | 0.803 |

Abbreviations: CI, confidence interval; AUC, area under the curve.
