# Supplementary material for: Development and validation of a nomogram for predicting 1-year mortality in infective endocarditis patients
Source: Front Cardiovasc Med. 2026 Mar 24;13:1730150. doi: 10.3389/fcvm.2026.1730150 (PMC13053318; doi:10.3389/fcvm.2026.1730150)
Supplement: Supplementary Table S2 — Proportional Hazards Assumption Test (Schoenfeld Residuals). [file Table2.docx]

**Supplementary Table S2. Proportional Hazards Assumption Test (Schoenfeld Residuals)**

Test of proportional hazards assumption for each covariate and globally using Schoenfeld residuals.

| Variable | Chi-square | df | P-value |
| --- | --- | --- | --- |
| Age | 0.373 | 1 | 0.541 |
| Embolism Symptoms | 0.180 | 1 | 0.672 |
| Heart Failure | 7.734 | 1 | 0.005 |
| Vegetation >10mm | 0.450 | 1 | 0.503 |
| Surgery | 1.216 | 1 | 0.270 |
| GLOBAL | 10.523 | 5 | 0.062 |

Note: The global test was non-significant (P=0.062), indicating the overall model satisfied the proportional hazards assumption. Heart failure showed a marginally significant individual test (P=0.005), suggesting potential time-varying effects for this covariate.
