# Supplementary material for: Development and validation of a nomogram for predicting 1-year mortality in infective endocarditis patients
Source: Front Cardiovasc Med. 2026 Mar 24;13:1730150. doi: 10.3389/fcvm.2026.1730150 (PMC13053318; doi:10.3389/fcvm.2026.1730150)
Supplement: Supplementary Table S3 — Bootstrap Validation Details (B=1000). [file Table3.docx]

**Supplementary Table S3. Bootstrap Validation Details (B=1000)**

Detailed results of 1,000-iteration bootstrap internal validation for the Cox proportional hazards model

| Metric | Original | Training | Test | Optimism | Corrected | Lower 95% CI | Upper 95% CI |
| --- | --- | --- | --- | --- | --- | --- | --- |
| Dxy (Somers' D) | 0.7576 | 0.7619 | 0.7477 | 0.0143 | 0.7433 | 0.6799 | 0.8043 |
| R² | 0.4601 | 0.4785 | 0.4501 | 0.0284 | 0.4317 | 0.3465 | 0.5018 |
| Slope | 1.0000 | 1.0000 | 0.9228 | 0.0772 | 0.9228 | 0.4945 | 1.2122 |
| D | 0.1740 | 0.1848 | 0.1689 | 0.0159 | 0.1581 | 0.1017 | 0.1987 |
| U | -0.0022 | -0.0022 | 0.0112 | -0.0134 | 0.0112 | -0.0124 | 0.1729 |
| Q | 0.1763 | 0.1871 | 0.1577 | 0.0293 | 0.1470 | -0.0262 | 0.2029 |
| g | 1.6779 | 1.8412 | 1.6578 | 0.1834 | 1.4945 | 0.5891 | 1.9292 |

Notes:

Dxy: Somers' Dxy rank correlation (C-index = (Dxy + 1) / 2)

R²: Nagelkerke R-squared

Slope: Calibration slope (ideal = 1.0)

D: Discrimination index

U: Unreliability index

Q: Overall quality index (D - U)

g: g-index (Gini's mean difference of linear predictor)

Original: Apparent performance in training data

Training: Mean performance in bootstrap samples

Test: Mean performance when bootstrap models applied to original data

Optimism: Training - Test (degree of overfitting)

Corrected: Original - Optimism (bias-corrected estimate)
